# Fertility preservation and subsequent reproductive outcomes in female pediatric patients with hematologic malignancies

**DOI:** 10.3389/fendo.2026.1791591

**Published:** 2026-05-15

**Authors:** Francesca Salvagno, Bernadette Evangelisti, Marta Agù, Roberta Aprile, Stefano Canosa, Carlotta Paschero, Erica Silvestris, Giacomo Corrado, Gianluca Gennarelli, Alberto Revelli

**Affiliations:** 1 Obstetrics and Gynecology 1U, Physiopathology of Reproduction and IVF Unit, Department of Surgical Sciences, Sant Anna Hospital, University of Turin, Turin, Italy; 2 Gynecologic Oncology Unit, IRCCS Istituto Tumori ‘Giovanni Paolo II’, Bari, Italy; 3 Gynecologic Oncology Unit, Department of Woman’s and Child Health and Public Health Sciences, Agostino Gemelli University Polyclinic, IRCCS, Rome, Italy; 4 Obstetrics and Gynecology 2U, Department of Surgical Sciences, Sant Anna Hospital, University of Turin, Turin, Italy

**Keywords:** fertility preservation, oncofertility, ovarian tissue cryopreservation, pediatric hematologic malignancies, premature ovarian insufficiency

## Abstract

Improved survival of pediatric and adolescent patients with hematologic malignancies has shifted attention toward long-term quality-of-life outcomes, including fertility. Female survivors of leukemia and lymphoma are at high risk of gonadal and uterine damage due to exposure to alkylating agents, radiotherapy, and hematopoietic stem cell transplantation, resulting in premature ovarian insufficiency, diminished ovarian reserve, and adverse pregnancy outcomes. Concurrently, fertility preservation strategies have evolved, expanding options for both prepubertal and postpubertal patients. This narrative review summarizes the impact of contemporary hematologic treatments on female reproductive function and critically appraises current fertility preservation approaches, including oocyte cryopreservation, ovarian tissue cryopreservation, ovarian transposition, and pharmacologic ovarian suppression. We also review evidence on spontaneous fertility and pregnancy outcomes in survivors and discuss the specific challenges associated with hematologic malignancies, such as treatment urgency and the risk of malignant cell reintroduction following ovarian tissue transplantation. Finally, emerging strategies, including artificial ovary construction and *in vitro* follicle growth, are briefly addressed. Integrating early, multidisciplinary oncofertility counseling into standard care is essential to optimize reproductive outcomes and ensure equitable access to fertility preservation for young cancer survivors.

## 1 Introduction

Survival rates among children and adolescents with cancer have steadily increased over the past decades. Currently, more than 80% of pediatric cancer patients—including those with hematological malignancies such as acute lymphoblastic leukemia (ALL), acute myeloid leukemia (AML), Hodgkin lymphoma (HL), and non-Hodgkin lymphoma (NHL)—achieve long-term survival (1, 2). Among adolescents and young adults (AYAs), survival has also improved; however, outcomes remain worse than those of younger children for most types of leukemia and lymphoma (3).

As survival rate improves, the focus of care has shifted from merely reducing mortality to enhancing quality of life, with fertility emerging as a major concern for patients and families (4, 5). Most young survivors express a strong desire for parenthood, and concerns about potential infertility contribute substantially to psychological distress. In female survivors, infertility risk is influenced not only by the direct gonadotoxic effects of therapy, but also by treatment-related disruption of pubertal development, uterine damage, and long-term endocrine dysfunctions (6, 7).

Hematologic malignancies represent nearly 40% of pediatric cancers and frequently require multimodal therapy, including alkylating chemotherapy, high-dose regimens for hematopoietic stem cell transplantation (HSCT), and, in some cases, abdominal or total body irradiation (TBI). All these are among the most gonadotoxic treatments in pediatric oncology (8, 9) and, as a consequence, survivors exhibit high rates of premature ovarian insufficiency (POI), significantly diminished ovarian reserve, and pregnancy-related complications compared with healthy peers (10–12).

On the other side, significant progress has been achieved in fertility preservation (FP) strategy. Established options such as oocyte cryopreservation are increasingly feasible for adolescent girls. Meanwhile, ovarian tissue cryopreservation (OTC) has transitioned from an experimental approach to a clinically accepted tool, particularly for prepubertal patients and those requiring urgent treatment (13–15). Dedicated oncofertility programs have expanded access to counseling and FP procedures, minimizing treatment delay and improving equity of care (16–18). Despite these progress, it is still an issue that female patients with hematologic malignancies remain less likely to be informed about fertility-related issues compared with other cancer populations (19).

FP in pediatric and adolescent patients implies multiple challenges. Time shortage is a major issue, as many hematologic cancers require immediate start of the therapy, limiting the feasibility of some FP interventions. Psychosocial complexity further complicates decision-making, as adolescents may have limited understanding of long-term infertility risk, and emotional stress can hinder their engagement in counseling. Ethical considerations arise as minors’ autonomy is still incomplete, and decisions involve parents and healthcare providers. Oncologic safety is another critical issue, particularly in hematologic malignancies, where the risk of reintroducing malignant cells in case of ovarian tissue re-transplantation is significant. Availability of FP procedures remains a very relevant problem, varying widely across countries and regions; to this regard, structured programs have been shown to substantially improve it (4). Economical cost is also relevant, as procedures, long-term storage, and follow-up care are expensive and often not covered by private insurance or public health system. Finally, prognostic uncertainty complicates counseling, as both the ovarian reserve of follicles and the susceptibility to treatment-related gonadotoxicity are highly variable and rather difficult to predict with certainty.

These factors underscore the need for early, multidisciplinary counseling and individualized FP planning aimed at optimizing outcomes in pediatric and adolescent patients with hematologic malignancies. FP strategies should be offered universally to eligible patients, alongside comprehensive psychosocial support throughout treatment and survivorship. This narrative review explores: i) the impact of today’s hematologic therapies on ovarian and uterine function; ii) the validated fertility preservation strategies in prepubertal and postpubertal girls; iii) the spontaneous fertility and pregnancy outcomes among survivors; iv) the reproductive outcomes following FP interventions; v) the emerging and experimental technologies.

## 2 Treatments for hematologic malignancies and effects on female fertility

### 2.1 Alkylating agents

Alkylating agents (e.g., cyclophosphamide, busulfan, procarbazine) are central in the treatment of hematologic malignancies and in chemotherapy regimens for hematopoietic stem cell transplantation (HSCT). Their gonadotoxic effects are dose- and age-dependent, with Cyclophosphamide Equivalent Dose (CED) models demonstrating a strong correlation between cumulative exposure and POI risk (20).These agents damage both growing and primordial follicles. Busulfan-containing regimens, frequently used in HSCT, are associated with extremely high risk of POI due to nearly complete follicular depletion (21). In case of ALL and AML, treatment intensification has reduced the use of the most gonadotoxic regimens; however, adolescents exposed to a high cumulative alkylating drug dose remain at relevantly increased risk of infertility (6).

### 2.2 Hematopoietic stem cell transplantation

HSCT involves infusion of healthy hematopoietic stem cells after high-dose chemotherapy, TBI, or both, to eradicate malignant cells and prepare the host for engraftment. HSCT is among the most detrimental interventions for female fertility. Pregnancy rates post-HSCT are reported to be very low (<5%) (22). POI prevalence is high, reaching 72.7–93.9%, depending on underlying disease, age, and medication regimen (13, 23). Age at transplantation significantly affects POI risk: younger girls (<13 years) exhibit lower POI rate than older adolescents (13). Meta-analytic data confirm higher infertility prevalence in females with malignant disease compared to benign conditions (24).

However, new evidence is currently emerging from the literature. The recent retrospective observational data from the French L.E.A. (Leucémies de l’Enfant et de l’Adolescent) program provides a critical long-term perspective on the reproductive trajectory of women treated for childhood leukemia. With a median follow-up of 18 years post-Hematopoietic Stem Cell Transplantation (HSCT), the study offers a evidence base for patient counseling. Despite the high gonadotoxic potential of HSCT conditioning—often involving Total Body Irradiation (TBI)—the study found that 12% (22 out of 178) of the women achieved at least one spontaneous pregnancy (25).

### 2.3 Radiotherapy

Radiotherapy gonadotoxicity depends on field, dose, and fractionation. Radiation Therapy (RT) significantly compromises reproductive health by depleting the ovarian follicle pool and impairing uterine and endometrial functionality. Direct ovarian irradiation (abdominal, pelvic, para-aortic) is highly toxic, with even low radiation dose (4–6 Gy) inducing permanent POI in adolescents (26, 27). The effective dose—the threshold at which 97.5% of patients experience immediate Primary Ovarian Insufficiency (POI)—is age-dependent, decreasing from 16 Gy at age 20 to 14 Gy at age 30. Beyond ovarian failure, RT induces dose-dependent uterine fibrosis, limiting the organ’s elasticity and distension capacity, which increases obstetric risks throughout pregnancy (28).

Cranial irradiation can disrupt hypothalamic–pituitary endocrine function. TBI is especially harmful, with single-fraction doses of 10–12 Gy conferring >80% POI risk (29, 30).

Radiation therapy during childhood, particularly before the onset of puberty, inflicts an extensive damage to reproductive potential. While adult women face functional decline, pre-menarchal girls face structural developmental arrest. It is confirmed a strong positive correlation between the patient’s age at the time of primary treatment and subsequent adult uterine volume. This relationship indicates that patients exposed to Total Body Irradiation (TBI) during early childhood are at the highest risk for severe uterine hypoplasia and irreversible structural dysfunction (31).

### 2.4 Targeted therapies and immunotherapy

Most targeted agents and immunotherapies used in pediatric hematology are not highly gonadotoxic compared with conventional chemotherapy. Nonetheless, cumulative effects with other treatments require careful evaluation (4).

### 2.5 Pubertal development interference

Hematologic diseases and treatment-related factors (malnutrition, need of corticosteroid support, etc.) can disrupt normal pubertal development, delaying sexual maturation (32).

Many survivors of onco-hematological malignancies develop endocrine late effects, including growth impairment and pubertal dysfunction, which may become evident months or even years after the completion of therapy (33). Recent clinical reviews highlight the high prevalence of neuroendocrine and gonadal late effects among long-term survivors of childhood cancer, with cohort studies reporting that up to 62 % of survivors experience at least one endocrine or reproductive late effect, and approximately one-third demonstrate clinically significant pubertal or gonadal dysfunction.

Antineoplastic therapies—including radiotherapy, high-dose chemotherapy, and prolonged corticosteroid treatment—can disrupt the hypothalamic–pituitary–gonadal (HPG) axis. Cranial radiotherapy involving the hypothalamic or pituitary regions remains a major risk factor for central endocrine dysfunction, with delayed puberty and hypogonadism recognized as late sequelae of hypothalamic–pituitary injury (34). In addition, the gonadotoxic effects of chemotherapy and radiotherapy increase the risk of primary gonadal dysfunction in childhood cancer survivors. Prolonged exposure to high-dose corticosteroids further contributes to endocrine dysregulation by altering hypothalamic–pituitary signaling and peripheral sex hormone homeostasis (34). Malnutrition and compromised nutritional status during and after cancer therapy represent additional significant contributors, as inadequate energy availability can delay activation of the HPG axis, which requires sufficient metabolic resources to sustain pubertal development and the pubertal growth spurt (35). The persistence and late onset of endocrine effects underscore the need for structured long-term follow-up in survivors of childhood onco-hematologic diseases. Consequently, international survivorship guidelines recommend periodic evaluation of growth parameters, pubertal staging, gonadotropins, sex steroids, and fertility markers throughout adolescence and early adulthood to allow timely identification and intervention for endocrine and reproductive dysfunction in this population.

## 3 Current fertility preservation strategies and critical issues

FP in pediatric hematology requires to find a balance among the urgency of therapy, the limited reproductive maturity, and all ethic/logistic complexities. Techniques include oocyte (and embryo) cryopreservation, OTC, ovarian transposition, and GnRH agonist-mediated ovarian suppression (**Table 1**).

**Table 1:** Fertility preservation techniques in female pediatric and adolescent patients with hematologic malignancies.

FP technique	Target population	Procedure/key steps	Outcomes	Limitations/considerations
Oocyte Cryopreservation (OC) *(established strategy)*	Postpubertal girls and adolescents	Controlled ovarian stimulation (COS), oocyte retrieval, vitrification	Post-thaw survival 70–90%; live-birth probability 25–40%	Requires ≥2 weeks for COS; procedural risks; ethical/legal issues; needs mature decision-making
Embryo Cryopreservation *(established strategy)*	Postpubertal girls with sperm source	COS, oocyte retrieval, fertilization, embryo freezing	High survival (95–98%); live-birth rates 60–70% in adults	Requires sperm; ethical/legal issues in minors; limited pediatric use
Ovarian Tissue Cryopreservation (OTC) *(established strategy)*	Prepubertal and postpubertal girls when urgent therapy required	Laparoscopic ovarian cortex harvesting, cryopreservation (slow freezing/vitrification)	>200 live births globally; endocrine function restoration in 90–95%	Risk of malignant contamination (esp. leukemia); graft longevity 2.5–5 years; experimental in prepubertal patients
Ovarian Transposition (Oophoropexy) (*established strategy)*	Girls receiving pelvic/abdominal radiotherapy	Surgical relocation of ovaries outside radiation field	Preserves endocrine function; spontaneous pregnancies rare	Does not protect from chemotherapy; surgical risks; ovarian migration possible
GnRH Agonists (Ovarian Suppression) *(established strategy)*	Postpubertal adolescents	Monthly or long-acting GnRHa during chemotherapy	May reduce risk of POI in some protocols	Ineffective against high-dose alkylators/TBI; does not preserve fertility for prepubertal girls
Artificial Ovary *(innovative strategy)*	High-risk patients (e.g., leukemia)	Follicle isolation, 3D scaffold encapsulation, transplantation	Preclinical: follicle survival, hormone production, oocyte maturation	Experimental; no clinical live births yet; scaffold optimization required
*In Vitro* Follicle Growth (IVFG) *(innovative strategy)*	Patients where autotransplantation unsafe	Activation of follicles *in vitro*, growth in 3D hydrogels, oocyte maturation	Preclinical/human early studies: follicle survival, limited meiotic progression	Long human folliculogenesis; endocrine regulation complex; no clinical pregnancies yet
Ovarian Stem Cell (OSC)–Based Neo-Folliculogenesis *(innovative strategy)*	High-risk patients with damaged ovarian tissue	OSC isolation, expansion, differentiation into oocyte-like cells, scaffold seeding	Experimental: oocyte-like cells with meiotic features	Highly experimental; tumorigenicity unknown; no clinical use
Ex Vivo Purging of Malignant Cells *(innovative strategy)*	Leukemia patients	Selective apoptosis of malignant cells in ovarian tissue prior to transplantation	Preclinical: preserves follicle morphology and function	Experimental; requires integration with artificial ovary or OTC; safety must be validated

### 3.1 Oocyte cryopreservation

Oocyte vitrification is well established in postpubertal girls and women: it was initially developed to preserve the fertility of cancer patients who wished to avoid the ethical concerns associated with embryo generation, particularly if performed in the absence of a partner (6, 12). Growing evidence supporting the efficacy and safety of female gamete vitrification has led both the American Society for Reproductive Medicine (ASRM) and the European Society for Human Reproduction and Embryology (ESHRE) to consider this technique as no longer experimental (36, 37).

The procedure involves controlled ovarian stimulation followed by transvaginal oocyte retrieval, typically requiring approximately two weeks. Post-pubertal girls below 16 years generally demonstrate a favorable response to ovarian hormonal stimulation, with very good oocyte yield, comparable or even higher than that observed in adults (12). Unfortunately, the need for urgent initiation of gonadotoxic therapy is a limitation for the application of this procedure. Moreover, the requirement of transvaginal oocyte retrieval in case of a young postpubertal girl raises psychological concerns.

The invasive nature of TV OR can provoke anxiety, embarrassment, and discomfort, particularly in adolescents with limited or no sexual experience. Many patients may experience feelings of violation, fear of pain, or distress related to genital exposure, which can interfere with procedural tolerance, engagement with care, and overall emotional well-being.

These psychological stressors are further compounded by anticipatory anxiety, concerns related to general anesthesia, and the presence of parents or clinical staff during counseling and procedural preparation. Addressing these challenges requires age-appropriate counseling, multidisciplinary support, and tailored psychological preparation (38).

When feasible, transabdominal (TA) retrieval offers an alternative that may reduce genital exposure and associated emotional distress, particularly in younger or prepubertal girls. However, TA retrieval is technically more challenging, may result in a lower oocyte yield, and still typically requires general anesthesia, which introduces additional psychological and safety considerations (39).Therefore, the choice between TV and TA retrieval must balance efficacy, patient age and pubertal status, psychological readiness, and procedural safety, while also considering cultural and family perspectives on reproductive interventions.

### 3.2 Embryo cryopreservation

Embryo cryopreservation is a well-established fertility preservation technique in adults; it relies on the availability of a sperm source and on the application of *in vitro* fertilization (IVF) technique. Its applicability in adolescents is very rare (4).

### 3.3 Ovarian tissue cryopreservation

**Figure 1:**
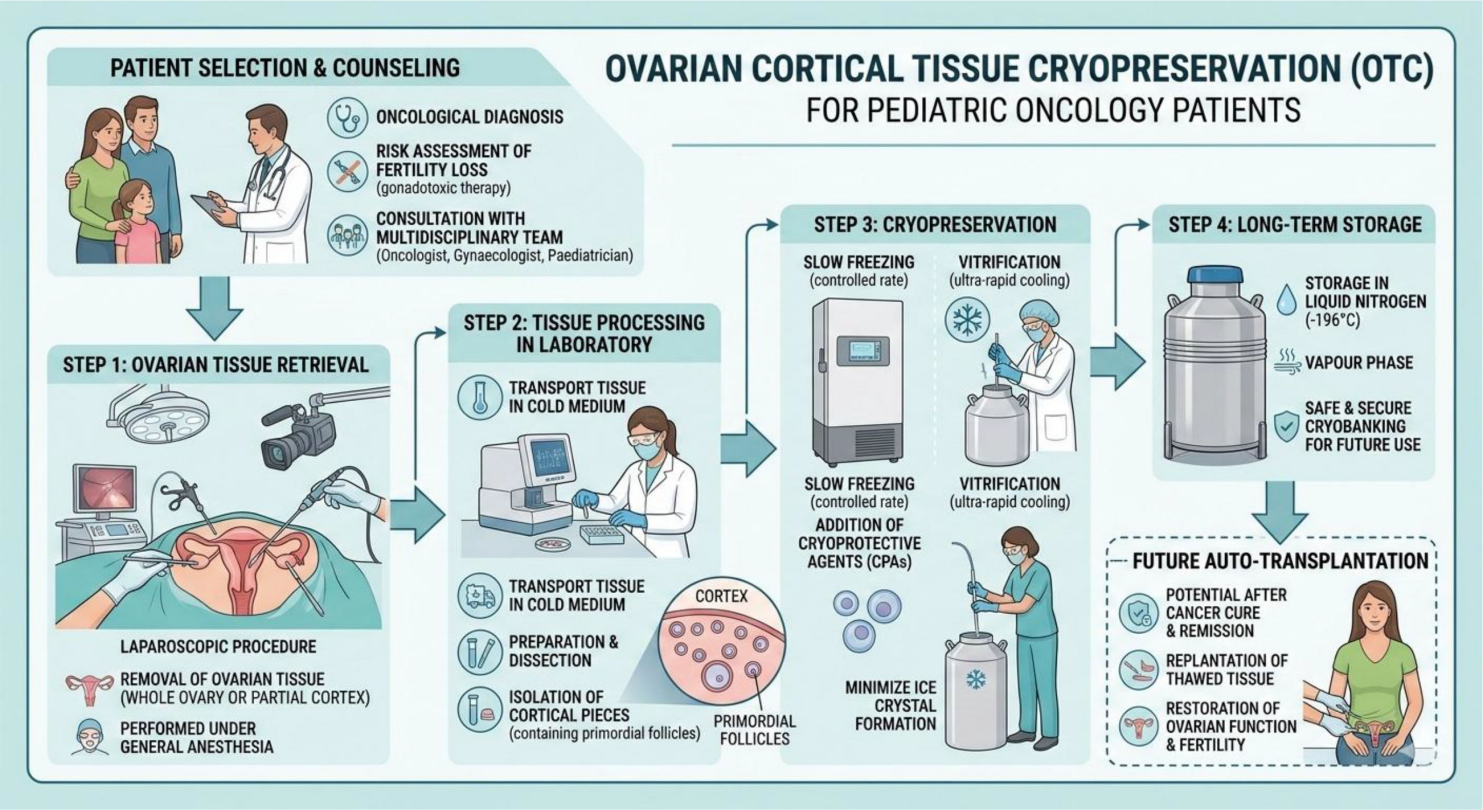
This image was created using generative AI tools.

The principal advantage of ovarian cortex cryopreservation is its feasibility, that is independent on the menstrual cycle phase and does not require a detailed assessment of ovarian reserve, as the cortical tissue consistently contains a substantial population of primordial follicles (**Figure 1**). Ovarian tissue retrieval is commonly performed laparoscopically, with approximately one third of each ovary excised. Using a sharp surgical blade, the medullary portion is carefully removed, and the remaining ovarian cortex is then divided into fragments measuring about 5 × 5 × 1–2 mm. These dimensions facilitate the rapid diffusion of cryoprotective agents (CPAs) into the tissue, thereby minimizing the damage caused by low temperatures to the follicular reserve. Although slow freezing has long represented the standard cryopreservation method, substantial follicular depletion and stromal cell injury have been documented (40). In an effort to improve freezing protocols, vitrification has been introduced as an alternative approach. This technique relies on ultra-rapid cooling in the presence of high concentrations of cryoprotectants and appears effective in limiting cellular damage by inducing a glass-like amorphous state, while also preserving stromal structure in a manner comparable to fresh tissue (41). Furthermore, Diaz-Garcia and colleagues assessed live birth rates after oocyte vitrification in comparison with ovarian tissue cryopreservation (OTC) followed by autologous transplantation in cancer patients undergoing gonadotoxic therapies. They reported that, although slightly higher success rates were observed with oocyte freezing, ovarian tissue vitrification should be regarded as a reliable alternative when oocyte cryostorage is not feasible (42). Notably, a recent systematic review and meta-analysis demonstrated a significantly higher proportion of intact stromal cells in vitrified tissue compared with slow-frozen samples, while no significant differences were found in terms of intact primordial follicle rates, DNA fragmentation levels, or mean primordial follicle density (43). Conversely, some studies have indicated that vitrification may lead to alterations in mRNA expression and delayed *in vitro* follicular growth in human ovarian tissue (44). More recently, the use of slush nitrogen has been proposed to further enhance vitrification efficiency. Compared with conventional liquid nitrogen, this approach appears to better preserve the morphology, ultrastructure, and viability of both follicles and stromal cells after thawing (45). Nevertheless, slow freezing remains the predominant technique worldwide for ovarian tissue cryopreservation, and to date only a few live births have been reported following vitrification procedures. Following thawing, ovarian tissue transplantation (OTT) can be performed at orthotopic sites, including the residual atrophic ovary, the broad ligament, the serosal surface of the Fallopian tube, or the pelvic wall at the ovarian fossa (46, 47).

The procedure may be carried out via laparoscopy, laparotomy, or microsurgical techniques, depending on surgeon expertise, patient anatomy, and the number of fragments to be transplanted. Laparoscopic approaches are minimally invasive, with smaller scars and faster recovery, whereas laparotomy may be preferred in cases of complex pelvic anatomy or when multiple grafts are required. Microsurgical techniques allow precise fixation of cortical fragments, optimizing revascularization and minimizing ischemic injury, which is critical for follicle survival. Comparative analyses suggest that laparoscopy is generally preferred in stable pediatric patients due to its minimally invasive nature, reduced postoperative pain, and faster recovery (48). OTT successfully restores ovarian function in most recipients. Pooled data indicate that ovarian activity, reflected by decreasing FSH and increasing estradiol, typically resumes within 3–6 months in approximately 70–95% of cases (48). Median graft function lasts around 2.5 years, with some cases exceeding seven years (49). Available reports suggest that prepubertal pediatric patients are capable of similar endocrine recovery, although published cases remain limited (50).Several hundred live births following OTC have been reported worldwide, with younger age at tissue explant being associated with higher follicle density and graft longevity (51). In hematologic malignancies, the risk of malignant contamination of the cryopreserved tissue limits the possibility of its reimplantation, prompting exploration of *in vitro* follicle maturation and tissue purging strategies (52).

Overall pregnancy rates following OTT are approximately 37%, with live birth rates around 28%, encompassing both spontaneous and assisted conceptions (48). Age at tissue retrieval remains a key determinant of reproductive outcomes, with younger patients generally achieving longer graft function and higher fertility potential. OTC is now recognized as a non-experimental fertility preservation option in the context of gonadotoxic therapy according to contemporary guidelines.

In pediatric patients, including prepubertal girls, OTC represents the only currently available fertility preservation strategy and can be offered when gonadotoxic treatment is imminent or when other established options, such as oocyte cryopreservation, are not feasible. Although it is no longer universally classified as experimental, its use in children remains highly specialized and should be performed in experienced centers with comprehensive counseling regarding the still limited long-term reproductive outcomes in strictly pediatric cohorts (53). In summary, OTC is now considered an acceptable and non-experimental technique for female patients, including prepubertal children, with specialized counseling advised regarding procedure risks, outcomes, and long-term fertility potential.

### 3.4 Ovarian transposition

Ovarian transposition relocates the ovaries outside the radiation field (4). Performed laparoscopically, ovaries are moved laterally or superiorly while maintaining vascular supply (26). The procedure protects against direct irradiation-induced gonadotoxicity, and carries low risk of torsion or ischemia (17).

However, pelvic radiotherapy is rarely used in the treatment of leukemia and lymphoma, as these malignancies are primarily managed with systemic chemotherapy and/or total body irradiation rather than localized pelvic fields. Pelvic irradiation is not routinely indicated in standard treatment protocols for leukemia and most lymphomas. Therefore, ovarian transposition is seldom indicated in this population and should be considered only when pelvic radiation is specifically planned (54). In cases where pelvic radiation is anticipated, ovarian transposition can be combined with ovarian tissue cryopreservation to maximize fertility preservation effectiveness.

### 3.5 Ovarian suppression with GnRH agonists

GnRH-agonists induce temporary ovarian quiescence, reducing follicular activity during chemotherapy (4, 5). This approach is ineffective in prepubertal girls, but may benefit post-pubertal adolescents facing urgent chemotherapy (7). The administration of GnRH-agonists during chemotherapy may reduce POI and preserve ovarian function, as measured by menstrual recovery and AMH/FSH levels (8).

Evidence supporting the fertility preservation (FP) benefit of GnRH agonists in pediatric hematologic malignancies is limited and remains controversial. Most data demonstrating ovarian protection derive from adult breast cancer cohorts, where temporary ovarian suppression during chemotherapy has been associated with reduced rates of premature ovarian insufficiency (55). Limitations in oncohematologic patients include the poor protective efficacy against high-dose alkylating agents or total body irradiation (TBI); thus, GnRH agonists are best used as part of a multimodal FP strategy, but should not be considered a replacement for established FP methods such as ovarian tissue cryopreservation (OTC) (53). In contrast, their role in menstrual suppression is well established. When initiated prior to treatment, they are effective in reducing or preventing menses, which is particularly important for quality of life and for minimizing bleeding complications in patients at risk of thrombocytopenia (56).

## 4 Spontaneous pregnancy and reproductive outcomes in survivors of pediatric hematologic malignancies

Spontaneous fertility after treatment for childhood and adolescent hematologic malignancies varies widely depending on age at treatment, cumulative gonadotoxic exposure, radiotherapy use, and need for hematopoietic stem cell transplantation (HSCT).

Survivors who received only standard-risk chemotherapy (without high cumulative doses of alkylating agents, pelvic irradiation, or HSCT) generally demonstrate a fairly good rate of spontaneous pregnancy compared to those exposed to high-dose alkylators, TBI/pelvic radiation, or HSCT. Cohort studies of survivors of acute lymphoblastic leukemia (ALL) showed variable fertility rates when gonadotoxic exposure was limited. In contrast, survivors undergoing HSCT, particularly with TBI, exhibited markedly reduced pregnancy rates. Recent large registry analyses confirm lower live birth rates after HSCT and after high cumulative alkylator exposure (57).

Resumption of menstruation and pubertal development does not necessarily imply a preserved fertility, as ovarian reserve may remain severely compromised and the apparent endocrine recovery can be temporary.

Cohort studies indicate that a substantial proportion of female survivors achieve spontaneous conception. Although overall pregnancy rates are lower than those in age-matched peers, many survivors who avoided high gonadotoxic exposures conceive naturally (58). In general, childhood cancer therapies do not significantly increase congenital anomalies or perinatal mortality in offsprings, although specific subgroups show higher risks of preterm birth and low birthweight (58, 59).

### 4.1 Likelihood of spontaneous pregnancy

The most comprehensive insights come from national cancer registries. Large population-based cohort studies report lower live birth rates compared to the general population, with substantial heterogeneity by diagnosis and treatment. Wallace et al. showed that women diagnosed before age 18 had significantly lower live birth rate by age 30 compared to the general population (37% vs. 58%), a pattern consistent across leukemia, Hodgkin lymphoma (HL), and non-Hodgkin lymphoma (NHL) (60). More recent treatment strategies show higher birth rate, reflecting lower gonadotoxicity and increased fertility preservation. The disease-specific outcomes are detailed below:

- Acute Lymphoblastic Leukemia (ALL): Modern ALL protocols that do not include HSCT carry a relatively low long-term infertility risk, as exposure to highly gonadotoxic alkylating agents is minimized. High-risk protocols including cyclophosphamide with cumulative doses (CED) >4,000 mg/m² are associated with increased POI. Many survivors treated without HSCT achieve spontaneous pregnancy, though population-based studies report slightly lower pregnancy and live birth rates compared to age-matched controls. The likelihood of natural conception is significantly lower after TBI or HSCT, emphasizing the importance of individualized fertility counseling and appropriate fertility preservation strategies (60, 61).
- Acute Myeloid Leukemia (AML): Fertility risk varies with treatment. Intensive cytarabine/anthracycline therapy is less gonadotoxic than alkylator-rich regimens or total/pelvic irradiation. Allogeneic HSCT with myeloablative conditioning carries the highest risk of permanent infertility (57). AML survivors treated with chemotherapy alone, particularly younger patients and those avoiding high-dose alkylators or pelvic irradiation, often retain sufficient gonadal function for spontaneous conception. In contrast, post-HSCT survivors experience high rate of POI and azoospermia, with substantially reduced natural conception probability (25).
- Hodgkin Lymphoma (HL): HL survivors generally retain a more favorable likelihood of spontaneous fertility than leukemia survivors, except when exposed to highly gonadotoxic regimens: intensified BEACOPP (bleomicyn, etoposide, adriamycin, cyclophosphamide, oncovin, procarbazine, prednisolone) or abdominal/pelvic radiotherapy. ABVD therapy (adriamycin, bleomycin, vinblastine, dacarbazine) is associated with minimal long-term gonadal impairment, whereas intensified BEACOPP causes substantial and often irreversible ovarian reserve depletion. Contemporary data confirm that ABVD-treated survivors often achieve spontaneous pregnancies at a rate close to the general population, whereas intensive therapies frequently require assisted reproductive techniques (62).

### 4.2 Impact of hematopoietic stem cell transplantation

HSCT is the most detrimental pediatric cancer treatment for fertility. Spontaneous pregnancy is biologically possible but uncommon, with age at transplantation and conditioning intensity as main determinants.

- Myeloablative with TBI or high-dose alkylators: Extensive primordial follicle loss, high POI rate, extremely low spontaneous pregnancy rate.
- Myeloablative without TBI, but high alkylator exposure: Substantial ovarian toxicity, partial recovery possible with occasional spontaneous pregnancies.
- Reduced-intensity conditioning (RIC): better ovarian reserve preservation, higher spontaneous puberty rate, and increased probability of later pregnancy, although still below population standard (63).

Age at transplantation modifies these outcomes: prepubertal recipients have higher chances of ovarian recovery than adolescents, but the risk of POI remains significant if TBI or high-dose alkylators are used (25).

### 4.3 Menstrual function and ovarian reserve

Gonadotoxic therapies, particularly alkylating agents, pelvic/TBI, and HSCT conditioning, accelerate follicle loss, causing menstrual irregularities and POI (64). Anti-Müllerian hormone (AMH) is the most sensitive marker for reduced ovarian reserve, correlating with lower antral follicle count: both parameters are lower in survivors compared to controls (65). Approximately one-third of female childhood-cancer survivors exhibit diminished ovarian reserve, with outcomes varying by treatment intensity, age at exposure, and fertility preservation intervention (66). Routine endocrine surveillance and individualized fertility counseling are recommended (67).

### 4.4 Pregnancy complications

Pelvic or abdominal radiotherapy, particularly following Total Body Irradiation (TBI) for hematologic malignancies, is a well-established cause of uterine factor infertility and subsequent high-risk pregnancies among childhood cancer survivors. The degree of risk is strongly influenced by the radiation dose and the irradiated uterine volume, with TBI conferring notably higher risks (68). Contemporary evidence, including studies by Legrand et al. (2025) (69), confirms significantly elevated obstetric risks in radiation-exposed survivors when compared with both the general population and those treated exclusively with chemotherapy. These risks primarily arise from direct damage to the uterine environment, increasing the likelihood of pregnancy loss, a finding supported by experimental data (68). The Dutch DCOG LATER-VEVO cohort has shown that radiation exposure correlates with a tenfold increase in preterm birth and a fifteenfold increase in low birth weight risk relative to matched controls. Additionally, large-scale survivor cohorts, such as the British Childhood Cancer Survivor Study (70) and the Nordic NOPHO cohort (71), report a 1.4–1.7-fold increased risk of miscarriage among women receiving uterine radiation doses >1 Gy. Furthermore, a 2025 meta-analysis by Lee et al. (72) highlights an increased burden of metabolic and hypertensive complications, including a 1.37-fold increased risk for preeclampsia and 1.29-fold for gestational diabetes in cancer survivors, particularly those exposed to abdominal or pelvic radiotherapy. These findings align with the observed structural deficits in the uterus, such as reduced uterine volume and impaired endometrial thickness, which contribute to obstetric complications (73). The risk is exacerbated by chemotherapy, particularly when combined with radiation as part of the conditioning regimen for hematopoietic stem cell transplantation (HSCT), which has been linked to severe obstetric outcomes, including preterm birth and fetal growth restriction (74).The mechanistic basis for these outcomes involves the combined effects of radiation and alkylating agents, which induce stromal fibrosis, endothelial injury, and impaired angiogenesis, thereby compromising uterine expansion and placental function (68). Collectively, these studies underscore the importance of individualized preconception counseling and specialized high-risk obstetric surveillance for this population, given the dose-dependent association between uterine irradiation and adverse pregnancy outcomes.

### 4.5 Psychosocial and behavior factors

Reproductive outcomes are influenced not only by biological effects, but also by psychosocial and behavior factors. Approximately 22% of female AYA survivors report voluntary childlessness, largely reflecting personal values rather than medical limitations (75). Over 20% of long-term survivors report persistent fertility-related concern, with distress and decision-making conflict reducing confidence in family-building (76). Fertility counseling should therefore include personalized discussion of values, reproductive goals, sexual identity, and life planning, to be performed ideally at diagnosis and throughout survivorship (77).

## 5 Outcomes of fertility preservation techniques in pediatric and adolescent patients with hematologic malignancies

Fertility preservation (FP) in pediatric and adolescent female cancer patients is defined not only by gamete or tissue survival but also by long-term reproductive outcomes, hormonal restoration, safety of reimplantation, and feasibility within urgent oncologic timelines. Increasing evidence supports the feasibility and clinical relevance of FP in childhood cancer survivors, particularly as survival rates improve.

### 5.1 Oocyte cryopreservation

Oocyte cryopreservation outcomes have substantially improved with vitrification, achieving post-warming survival rates of 70–90% and fertilization/blastocyst formation comparable to fresh oocytes (78). Success depends primarily on age at cryopreservation and number of stored oocytes, with cumulative live birth probability ranging from 25–40% (79). Better outcomes are observed in patients who bank ≥15–20 mature oocytes. Despite these promising outcomes, the utilization rate of cryostored oocytes remains low, typically <30% at 5–10 years, emphasizing the importance of realistic counseling regarding FP expectations (80).

#### 5.1.1 Pediatric oocyte cryopreservation

- Post-pubertal adolescents: The median yield is averagely of 10–15 mature oocytes, depending on age, ovarian reserve (AMH, AFC), disease type, and timing relative to chemotherapy (20, 81).
- Prepubertal girls: Oocyte cryopreservation is not possible; ovarian tissue cryopreservation (OTC) is the only option.
- Hematologic malignancy patients: Baseline ovarian reserve may be reduced, but double stimulation or random-start protocols can be applied in order to achieve adequate oocyte yield (82, 83). Post-warming survival and blastocyst formation rates appear to be high (~73–92%), but pediatric-specific outcome data are very limited (6).

#### 5.1.2 Pregnancy outcomes from adolescent oocytes

The quite limited amount of available data suggests that oocytes cryopreserved during adolescence maintain developmental competence, with post-warming survival >85%, fertilization rate 70–80%, and blastocyst formation rate 40–50% (20). Return rate remains low (≤5–30%), limiting the number of observed pregnancies. Cohort follow-up suggests that eventual live births are achievable, with a rate of 25–40% among patients returning to claim the use of their oocytes (84).

### 5.2 Embryo cryopreservation

Embryo cryopreservation, typically via vitrification, is infrequently used in case of adolescents. Post-warming blastocyst survival reaches 95–98%, with very good implantation and pregnancy rate (85–87). Evidence in adolescents with hematologic malignancies is limited, and outcomes are generally extrapolated from adults (88).

### 5.3 Ovarian tissue cryopreservation

OTC is the only established FP option for prepubertal girls and an essential strategy for adolescents requiring urgent therapy that precludes ovarian stimulation. Re-grafting of ovarian cortex reliably restores endocrine function in most recipients (~90–95%) within 3–6 months, with graft function persisting for 2.5–5 years on average, and occasionally beyond a decade (89, 90).

#### 5.3.1 Live births

Global experience demonstrates >250 live births post-OTC re-grafting, with pregnancy rates of 30–40% per transplantation and live-birth rates per patient of 25–30% (higher in those who cryopreserved at younger age). Successful pregnancies have been reported from tissue harvested in childhood (90, 91).

#### 5.3.2 Hematologic malignancy considerations

Leukemia patients are at high risk of ovarian tissue contamination with malignant cells, particularly ALL, AML, and blast-phase CML, limiting re-grafting safety (92). Investigational strategies—tissue purging, ex vivo follicle isolation, and *in vitro* folliculogenesis—aim to mitigate this risk but remain experimental.

### 5.4 Ovarian transposition (oophoropexy)

Ovarian transposition can preserve endocrine function by relocating ovaries outside radiation fields. Hormonal preservation is reported in a substantial fraction of pediatric and adolescent patients, though spontaneous pregnancies are rare and mostly documented in case reports or mixed-cohort studies (93, 94). Complications include ovarian cysts, migration, and difficulties in later oocyte retrieval. Combined approaches (e.g., ovarian transposition plus OTC) are recommended when feasible.

### 5.5 Gonadotropin-releasing hormone agonists

GnRHa co-treatment is aimed at reducing chemotherapy-induced ovarian failure; it lacks robust evidence in pediatric/adolescent hematologic malignancies. Retrospective studies in HSCT recipients show no significant protection of ovarian reserve (95, 96). Probably GnRHa cannot prevent follicle loss from high-dose alkylating agents or total-body/pelvic irradiation. Guidelines advise their use only as an adjunct or when established cryopreservation methods are not feasible (97).

## 6 Counseling

### 6.1 Counseling before treatment

International guidelines emphasize that fertility preservation is a time-sensitive priority in oncology. All clinicians are tasked with initiating discussions on treatment-related infertility immediately following diagnosis. The literature supports a “strong recommendation” for early intervention, advocating for the referral of all patients to multidisciplinary fertility teams. This approach ensures that individualized preservation strategies—tailored to age, treatment type, and patient preference—can be implemented without compromising the oncology timeline. Moreover, clinicians should promote patient participation in clinical registries to further refine the understanding of gonadotoxic risks and the long-term efficacy of preservation interventions. A multidisciplinary support framework, comprising genetic counselors and mental health practitioners is essential to mitigate the multifaceted psychosocial and economic obstacles to fertility preservation (28).

### 6.2 Endocrine surveillance and survivorship care

Cancer survivors are at increased risk of gonadal dysfunction and impaired fertility due to exposure to alkylating agents, platinum compounds, hematopoietic stem cell transplantation (HSCT), and pelvic or total body irradiation (TBI). Data from large cohort studies, including the Childhood Cancer Survivor Study (CCSS), demonstrate that female survivors have a significantly increased risk of premature ovarian insufficiency (POI), delayed puberty, and reduced ovarian reserve compared with sibling controls (98, 99).Follow-up should be risk-adapted, longitudinal, and coordinated between pediatric oncology and pediatric/adolescent endocrinology, as recommended by survivorship guidelines. A structured surveillance pathway improves early detection of ovarian dysfunction and ensures timely fertility counseling. Risk stratification should be based on: Alkylating agent dose (cyclophosphamide equivalent dose), radiation field and dose, HSCT exposure and age at treatment.

High-risk patients require earlier and more intensive endocrine evaluation (99–101).The frequency of follow up should be modulated according to the age of the patient. During childhood (prepubertal years) annual clinical assessment is recommended for all at-risk survivors. Evaluation should include growth velocity, BMI, pubertal staging (Tanner), and review of treatment exposure (102). Patients exposed to high-risk therapies (e.g., HSCT with TBI, high cumulative alkylator dose, ovarian/pelvic irradiation ≥10–15 Gy) may benefit from biochemical screening beginning in mid-childhood (98, 99). During expected pubertal age (girls: 8–13 years), follow-up should occur every 6–12 months. Increased frequency (every 6 months) is appropriate when there is absent breast development by age 13, pubertal arrest, or clinical suspicion of estrogen deficiency (102, 103). During Adolescence and early adulthood annual review is recommended in asymptomatic patients. More frequent evaluation is indicated in the presence of menstrual irregularities, amenorrhea, or biochemical evidence of declining ovarian reserve (101). Surveillance should integrate clinical, biochemical, and imaging parameters, tailored to age and pubertal stage. Hormonal evaluation should include blood measurement of follicle-stimulating hormone (FSH), Luteinizing hormone (LH), Estradiol (E2) and Anti-Müllerian hormone (AMH). AMH is a sensitive marker of ovarian reserve and correlates with cumulative gonadotoxic exposure in childhood cancer survivors (101, 102). However, AMH should not be used in isolation to diagnose POI, particularly in early adolescence (103, 104).

Pelvic ultrasound can be useful to assess uterine size, ovarian volume and antral follicle count (when feasible. The evaluation should include Auxologic and pubertal monitoring: height and growth velocity, tanner staging, bone age (if delayed puberty suspected). HRT should be initiated in cases of confirmed hypergonadotropic hypogonadism or absent/delayed puberty attributable to ovarian insufficiency. The diagnostic criteria for POI in adolescents are amenorrhea or absent puberty, elevated FSH on two measurements (>25 IU/L, ≥4 weeks apart), low estradiol.

Early estrogen replacement is critical for bone mineral accrual, cardiovascular protection, and psychosocial development (104, 105). Survivorship cohort data demonstrate that untreated ovarian insufficiency is associated with reduced peak bone mass and increased long-term morbidity (105).

When spontaneous puberty does not occur, stepwise estrogen induction is recommended:

1. Low-dose 17β-estradiol (oral or transdermal)
2. Gradual dose escalation every 6 months
3. Addition of cyclic progesterone after breakthrough bleeding or after 12–24 months of estrogen therapy

Gradual pubertal induction over approximately 2–3 years mimics physiologic puberty and optimizes uterine and skeletal development (105). Transdermal estradiol may provide more physiologic hormone levels and reduced thrombotic risk compared with oral preparations (103).

## 7 Innovative and emerging technologies in fertility preservation

While ovarian tissue re-transplantation (OTT) is an established FP strategy, it carries a substantial risk of disease reintroduction in patients with hematologic malignancies (106, 107). In leukemias and aggressive lymphomas, ovarian cortex may harbor occult malignant cells even when histology is negative, rendering re-grafting potentially unsafe. These limitations have stimulated the development of innovative FP strategies designed to preserve fertility while minimizing risk. Current major avenues of research include the artificial ovary, *in vitro* folliculogenesis and *in vitro* follicle growth (IVFG), stem-cell–based neo-folliculogenesis, and ex vivo purging of malignant cells.

### 7.1 The artificial ovary

The development of an artificial ovary (AO) is intended to re-establish the three-dimensional ovarian architecture, thereby promoting follicular survival and growth, supporting physiological sex steroid secretion, and ultimately enabling the generation of fertilization-competent mature oocytes, while reducing the risk of cancer recurrence (108, 109). In brief, AO technology relies on the isolation of follicles from the ovarian cortex—either fresh or thawed following cryopreservation—and their subsequent encapsulation within a bioengineered matrix. This approach is designed to maintain follicular viability and, after transplantation of the AO into the patient, to ensure functional restoration, including both gametogenic activity and endocrine function. The process typically involves three stages:

1. Cryopreservation of ovarian cortex prior to gonadotoxic therapy, using slow freezing or vitrification optimized to preserve primordial follicles (108).
2. Follicle isolation from thawed tissue via mechanical and enzymatic digestion, followed by repeated washing to remove stromal and hematologic contaminants (109).
3. Encapsulation of isolated follicles within a three-dimensional (3D) scaffold, sometimes supplemented with stromal or endothelial cells, for subsequent orthotopic or heterotopic transplantation (106).

This approach leverages the natural separation of follicles from vascular and stromal compartments by the basement membrane and basal lamina, reducing the likelihood of co-transplanting malignant cells. Follicle suspensions can also be screened for minimal residual disease using sensitive molecular or flow-cytometric assays prior to scaffold incorporation (107).

Scaffold selection is critical. Ideal biomaterials are biocompatible, minimally immunogenic, allow nutrient and oxygen diffusion, preserve 3D follicle architecture and oocyte–granulosa communication, and degrade in a controlled manner to permit vascularization (108).

- Natural polymers: Collagen and plasma clots were first used due to biocompatibility, with murine studies demonstrating growth to preovulatory stages and viable offspring post-IVF. Plasma clots are limited by compositional variability and rapid degradation.
- Fibrin gels: pro-angiogenic properties support rapid vascularization and follicle survival. Supplementation with VEGF or platelet lysate enhances outcomes.
- Alginate: Maintains follicle spherical architecture and oocyte–granulosa cross-talk; widely used for 3D follicle culture and encapsulated IVFG (110).
- Synthetic polymers (PEG): Allow precise control of stiffness, porosity, degradation, and functionalization with adhesive motifs or growth factors; hybrid scaffolds combine mechanical robustness with biological cues.
- Advanced engineering: 3D printing and microfabrication enable “ovary-like” scaffolds with controlled pore size, follicle distribution, and vascularization, representing the frontier of preclinical ovarian tissue engineering (97).

### 7.2 *In vitro* folliculogenesis and *in vitro* follicle growth

*In vitro* folliculogenesis seeks to generate mature oocytes entirely ex vivo, avoiding transplantation and oncologic risk (110, 111). The process consists of two phases:

1. Early follicle growth within ovarian tissue: Cortical fragments are cultured to activate primordial follicles and promote progression to primary and secondary stages while preserving the stromal microenvironment.
2. Isolated follicle culture: Developed follicles are mechanically separated and transferred to 3D hydrogels (e.g., alginate, fibrin, PEG-based scaffolds) to support growth to the antral stage, steroidogenesis, and acquisition of meiotic competence.

IVFG in murine models enables the production of MII oocytes, which can be fertilized to yield live offspring (110). Human translation faces challenges due to longer folliculogenesis, larger follicle size, and complex endocrine regulation. Early human studies demonstrate follicle survival, growth, and limited meiotic progression *in vitro*, suggesting potential for clinical application in patients for whom transplantation is contraindicated. The network analyses of over 500 studies by Bernabò et al. (2021) (112), reveals that key regulators of folliculogenesis include PI3K, KIT ligand, JAK–STAT, SMAD4, cAMP, and metabolic regulators like mTOR/FOXO and VEGF. Optimizing these pathways, alongside biomaterials and culture conditions, is expected to improve IVFG efficiency.

### 7.3 Stem-cell–based neo-folliculogenesis

Experimental studies in animal models have suggested an active contribution of stem cells to the restoration of ovarian function following human ovarian tissue (OT) grafting. In this context, increasing attention has been directed toward the potential involvement of resident oogonial stem cells (OSCs) in the resumption of ovarian activity (113, 114). OSCs, also referred to as germline stem cells, have been identified within the ovarian surface epithelium of mice, where they represent a very small fraction of the total cell population (approximately 0.014%) and become progressively less abundant with advancing age (115). A growing body of evidence supports the hypothesis that reintroduction or survival of OSCs within transplanted OT after chemotherapy may contribute, at least in part, to the recovery of ovarian function. Nevertheless, the identification and validation of transferable “oogenic” factors capable of driving OSC differentiation into functional oocytes remain an area of active investigation. Despite these limitations, the demonstrated capacity of OSCs to generate oocyte-like cells *in vitro* in mammalian models provides a strong rationale for high-throughput screening of candidate oogenic factors, which can subsequently be rigorously evaluated for their ability to expand the ovarian reserve *in vivo* (116, 117). In humans, under appropriate *in vitro* culture conditions, Ddx4-positive OSCs isolated from both non-menopausal and menopausal women have been shown to differentiate into large haploid oocyte-like cells expressing key oocyte markers, including growth differentiation factor 9 (GDF-9) and synaptonemal complex protein 3 (SYCP3); notably, these cells were also capable of entering meiosis (118, 119).

Potential applications in oncofertility include: i) expansion of OSCs from post-therapy ovarian tissue; ii) seeding into decellularized ECM or artificial scaffolds to regenerate a “neo-ovary”. However, the field remains highly experimental due to limited lineage-tracing evidence, potential overlap with somatic or malignant cells, and unknown tumorigenicity.

### 7.4 Ex vivo purging of malignant cells

In hematologic malignancies, ovarian tissue may harbor malignant cells, precluding safe re-grafting. Reported contamination rates are ~12% in prepubertal ovaries and 37% in prepubertal testes (85). Even minimal residual disease can trigger relapse, necessitating targeted safety interventions. Ex vivo purging strategies exploiting the differential biology of malignant and ovarian cells (120) demonstrated that treating human ovarian cortex contaminated with AML/CML cells with the Aurora B/C kinase inhibitor GSK1070916 induced selective apoptosis of leukemic cells while preserving primordial follicle morphology, glucose uptake, and *in vitro* growth. Combining purging with follicle isolation and artificial ovary construction could extend FP to high-risk patients.

## 8 Conclusion

Advances in pediatric and adolescent hematologic malignancy care have shifted clinical priorities toward quality-of-life outcomes, including fertility. While established techniques (oocyte/embryo cryopreservation for post-pubertal adolescents and ovarian tissue cryopreservation for prepubertal children) offer real hope, re-grafting of cryostored ovarian tissue carries oncologic risk in leukemias and some lymphomas. Emerging technologies, including the artificial ovary, IVFG, and OSC-based neo-folliculogenesis, promise safe fertility restoration in high-risk populations. The success of FP depends not only on biological innovation but also on integrated, multidisciplinary oncofertility programs ensuring timely individualized counseling, psychosocial support, and equitable access. Challenges remain in standardization, long-term safety evaluation, ethical oversight, and demonstration of clinical efficacy. Nonetheless, the trajectory of research suggests a future where fertility restoration is achievable even in patients with malignancies previously considered contraindicated for tissue-based FP, ensuring to young survivors the possibility of biological parenthood.

## Glossary

ABVD: Adriamycin, Bleomycin, Vinblastine, Dacarbazine
AFC: Antral Follicle Count
ALL: Acute Lymphoblastic Leukemia
AMH: Anti-Müllerian Hormone
AML: Acute Myeloid Leukemia
AO: artificial Ovary
ASCO: American Society of Clinical Oncology
ASRM: American Society for Reproductive Medicine
AYA: Adolescents and Young Adults
BEACOPP: Bleomycin, Etoposide, Adriamycin, Cyclophosphamide, Oncovin, Procarbazine, Prednisolone
cAMP: cyclic adenosine monophosphate
CED: Cyclophosphamide Equivalent Dose
CML: Chronic Myeloid Leukemia
ESHRE: European Society for Human Reproduction and Embryology
FGR: Fetal Growth Restriction
FP: Fertility Preservation
FSH: Follicle-Stimulating Hormone
FOXO: Forkhead box class O (proteins)
GDF-9: growth differentiation factor 9
GnRH/GnRHa: Gonadotropin-Releasing Hormone/GnRH Agonists
HL: Hodgkin Lymphoma
HSCT: Hematopoietic Stem Cell Transplantation
IVF: *In Vitro* Fertilization
IVFG: *In Vitro* Follicle Growth
JAK: Janus Kinase
mTOR: mechanistic Target of Rapamycin
NHL: Non-Hodgkin Lymphoma
OC: Oocyte Cryopreservation
OTC: Ovarian Tissue Cryopreservation
OTT: Ovarian Tissue Transplantation
OSCs: oogonial stem cells
PEG: polyethylene glycol
PI3K: phosphatidylinositol 3-kinase
POI: Premature Ovarian Insufficiency
RIC: Reduced-Intensity Conditioning
RT: Radiation Therapy
SGA: Small-for-Gestational-Age
SYCP3: synaptonemal complex protein 3
TBI: Total Body Irradiation
VEGF: vascular endothelial growth factor
